# Stylomandibular False Ankylosis: An Unusual Complication After Vascularized Iliac Crest Flap for Mandibular Reconstruction

**DOI:** 10.7759/cureus.37615

**Published:** 2023-04-15

**Authors:** Bruno Morgado, Filipa Poleri, Carolina Gaspar, Horácio Costa, Horácio Zenha

**Affiliations:** 1 Plastic and Reconstructive Surgery, Centro Hospitalar de Vila Nova de Gaia/Espinho, Vila Nova de Gaia, PRT; 2 Faculty of Medicine and Biomedical Sciences, University of Algarve, Faro, PRT

**Keywords:** mandible, ankylosis, styloid, ameloblastoma, iliac crest

## Abstract

Stylomandibular fusion is a poorly documented and rare complication of maxillofacial surgical procedures. This case report describes a patient presenting with stylomandibular false ankylosis following mandibular reconstruction. A 59-year-old female patient underwent segmental mandibular resection and reconstruction for a defect resulting from ameloblastoma surgery using an iliac crest free flap. A styloid fracture was detected postoperatively, and the patient was managed conservatively. In the third postoperative year, the patient presented with marked limitation of oral gape. A diagnosis of stylomandibular false ankylosis was made, and the patient underwent an ostectomy of the aberrant bone, with improved mouth opening. The abnormal union between the styloid process and the mandible is a previously unreported complication in the use of iliac crest free flaps. This case report emphasizes the importance of being vigilant for stylomandibular false ankylosis, especially when there is a restriction of oral aperture postoperatively following reconstructive procedures involving bone flaps.

## Introduction

The iliac crest free flap is a versatile and reliable method for autologous mandibular reconstruction for medium and large defects. This flap can be composed of bone, soft tissue, and skin, has a natural contour for mandibular reconstruction, provides ample vertical and horizontal bone height, and allows dental rehabilitation. It is, therefore, an ideal option for complex mandibular reconstruction [1]. A few complications of this procedure are described, with hardware plate exposure and flap wound infections being the most common microvascular bone flap transfer. Rarer complications include fistula, wound dehiscence, bone exposure, cutaneous perforation, flap anastomotic revision or failure, and heterotopic bone formation [2,3].

Stylomandibular fusion is a rare condition characterized by an abnormal union between the styloid process and the lower jaw and, to our knowledge, is unreported to date as a complication of iliac crest free flap for mandible reconstruction. Many cases of calcified stylohyoid ligaments have been reported, and few report on ankylosis of the temporomandibular joint due to heterotopic bone formation or stylomandibular ligament calcification [4,5].

We present a case of stylomandibular false ankylosis due to aberrant bone formation between the styloid process and a mandibular iliac crest flap, following the reconstruction of a mandibular defect resulting from ameloblastoma surgery.

## Case presentation

**Figure 1:**
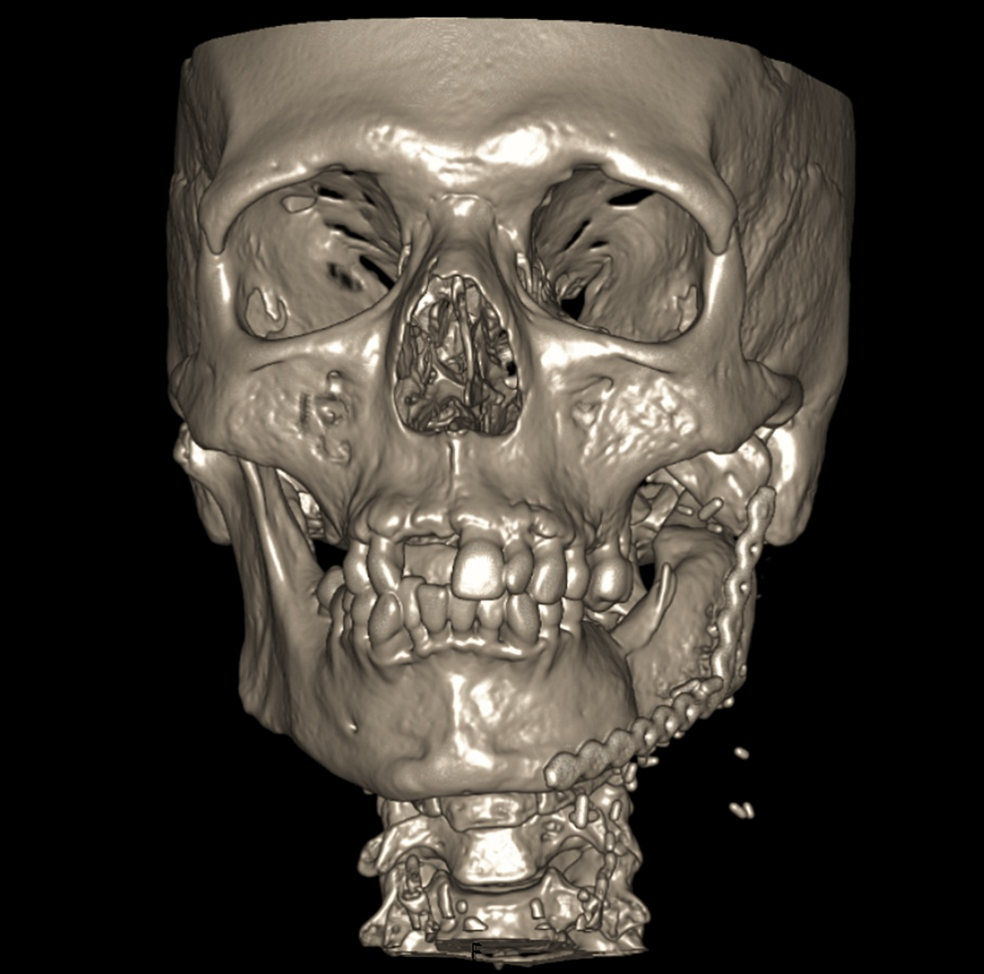
CT bone window with 3D reconstruction Displays mandibular reconstruction using a free iliac crest flap after ameloblastoma resection.

A 59-year-old female diagnosed with mandibular ameloblastoma involving the left body and ramus was referred to our clinic for surgical treatment. The patient received treatment through segmental mandibular resection and reconstruction, which involved microsurgical free tissue transfer of a right iliac crest flap. The procedure was planned using CT 3D biomodelling and included end-to-end anastomosis to the left facial artery and left tributary of the internal jugular vein (Figure 1).

**Figure 2:**
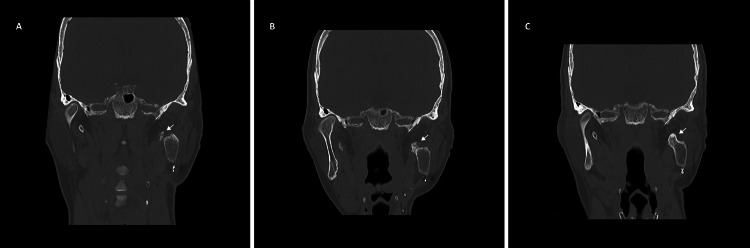
Postoperative coronal CT bone window (A) Minimally displaced fracture in the distal third of the left temporal styloid fracture, in proximity with the bone flap (white arrow). (B) Pseudoarthrosis of the left temporal styloid process in conjunction with the bone flap and concomitant hypertrophy of the bone fragments (white arrow). (C) Complete fusion of the left temporal styloid process with the bone flap (white arrow).

The postoperative period was uneventful. A control CT examination performed two months postoperatively demonstrated a minimally displaced fracture of the left styloid process, which was managed conservatively (Figure 2A). The patient complained of residual postoperative pain but showed good interincisal aperture and no other signs of complications. She maintained regular follow-ups at the outpatient clinic and, in the second postoperative year, reported an increase in facial pain that was exacerbated by masticatory movement, accompanied by a slight limitation of oral aperture. CT showed pseudoarthrosis between the bone flap and the left styloid process with hypertrophy of the styloid process (Figure 2B). The patient was managed conservatively with a regimen comprising exercises to increase oral gape, analgesics, botulinum toxin injections, and mesotherapy utilizing thiocolchicoside and piroxicam, resulting in some improvement of symptoms. In the third postoperative year, the patient returned for a follow-up appointment, presenting with complaints of exacerbated facial pain, trismus, and a marked limitation of oral gape, resulting in difficulties with adequate food intake. CT showed aberrant ossification/false ankylosis between the left styloid process and the ipsilateral mandibular bone flap (Figure 2C).

**Figure 3:**
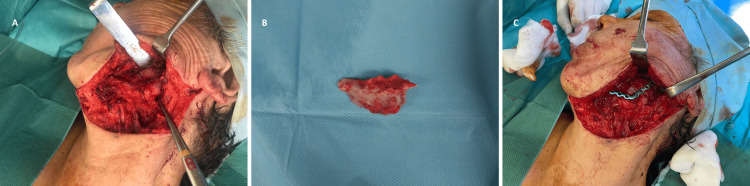
Ostectomy of the abnormal bone fragment (A) Lateral view of the patient following a left submandibular surgical approach and removal of the reconstruction plate. The blue mark indicates the lower osteotomy line of the aberrant bone fragment. (B) The heterotopic bone fragment was removed. (C) Lateral view after replacement of the reconstruction plate and filling of the defect with a hemostatic agent.

The patient was scheduled for surgery, and an ostectomy of the aberrant bone fragment was performed utilizing a piezoelectric saw (Figure 3).

**Figure 4:**
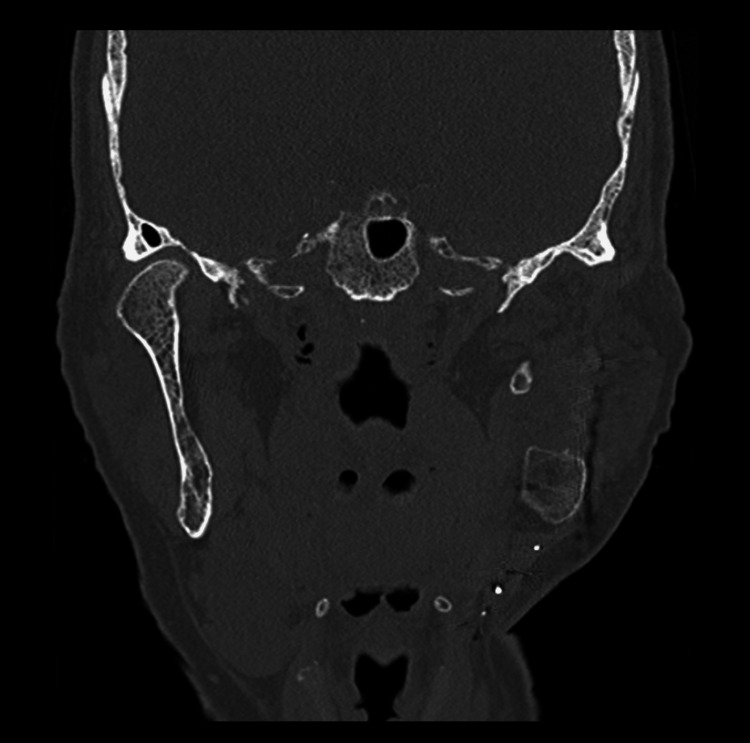
Coronal CT bone window after revision surgery Illustrates complete removal of the bone fragment, with no evidence of new bone growth between the remaining portion of the styloid process and the bone flap.

There were no complications during the recovery period, and she was released from the hospital two days later. Outpatient follow-up was uneventful with the patient experiencing reduced facial pain and a significant increase in the interincisal opening (42 mm). Control CT at one-year follow-up showed complete resolution of the false ankylosis, and the patient remains symptom-free to date (Figure 4).

## Discussion

False ankylosis, or aberrant ossification between the styloid process and mandibular bone flaps, is an unreported complication of skeletal reconstruction following tumor ablation surgery. Most case reports describe a fusion of the styloid process to the mandible as a consequence of stylomandibular ligament calcification [5]. A fusion of the styloid process with the mandible is believed to be related to its strong ability to form bone, the repositioning of the mandible after bilateral orthognathic surgery, past surgical injury, and in some cases, prolonged irritation of the stylomandibular ligament [6].

In this case report, it appears that the primary cause of heterotopic ossification was a closed fracture of the styloid process that occurred during ameloblastoma resection, given that the placement of the bone flap is typically non-traumatic. This resulted in an abnormal consolidation to the internal, subcortical surface of the iliac crest bone flap. Fractures of the styloid process are relatively uncommon among individuals who have sustained traumatic injuries and are more frequently observed in conjunction with fractures of other facial bones, particularly the mandible [7]. It should be noted that the occurrence of this condition in nonsyndromic individuals without Eagle's syndrome is relatively uncommon, as is the case in our patient [8].

A contributing factor for stylomandibular fusion is noncompliance with mouth-opening exercises designed to separate contacting bone surfaces [5]. Our patient displayed poor adherence to these exercises as a result of postoperative pain, which may have exacerbated the abnormal ossification process.

Treatment for stylomandibular fusion is generally considered to be surgical, with careful removal of the heterotopic bone fragment [5]. It is advisable to utilize a piezoelectric saw in ostectomy as there is evidence to suggest that it may decrease the likelihood of heterotopic bone formation [9].

## Conclusions

In conclusion, stylomandibular aberrant ossification is a possible complication of bony microsurgical free tissue transfer in maxillofacial reconstruction, as a consequence of styloid fracture during surgical manipulation. Care should be taken to prevent injury to the styloid process during the surgical reconstruction of the mandible. In the unlikely event of an intraoperative detection of a fracture, an ostectomy of the fractured styloid process or the utilization of an interface, such as a fascial graft or dermal regeneration template, may be considered as a means of preventing this complication. Care should be taken during bone fragment resection as to avoid injury to the carotid vessels.

Surgeons should exercise a high degree of vigilance for stylomandibular false ankylosis, particularly in instances where there is a limitation of oral aperture postoperatively, following maxillofacial reconstructive procedures that involve the utilization of bone flaps.

## References

[1] Sönmez E, Tözüm TF, Tulunoglu I, Sönmez NS, Safak T (2012). Iliac crest flap for mandibular reconstruction after advanced stage mandibular ameloblastoma resection. Ann Plast Surg.

[2] Ritschl LM, Mücke T, Hart D, Unterhuber T, Kehl V, Wolff KD, Fichter AM (2021). Retrospective analysis of complications in 190 mandibular resections and simultaneous reconstructions with free fibula flap, iliac crest flap or reconstruction plate: a comparative single centre study. Clin Oral Investig.

[3] Knitschke M, Siu K, Bäcker C, Attia S, Howaldt HP, Böttger S (2020). Heterotopic ossification of the vascular pedicle after maxillofacial reconstructive surgery using fibular free flap: introducing new classification and retrospective analysis. J Clin Med.

[4] Guttu RL, Laskin DM (1987). False ankylosis from fusion of the styloid process to the mandible after orthognathic surgery. J Oral Maxillofac Surg.

[5] Agarwal B, Mohod M, Bhutia O, Roychoudhury A (2016). Stylomandibular fusion complicating recurrent bilateral temporomandibular joint ankylosis. Br J Oral Maxillofac Surg.

[6] EA WW (1958). Elongated styloid process; symptoms and treatment. AMA Arch Otolaryngol.

[7] Tiwary P, Sahoo N, Thakral A, Ranjan U (2017). Styloid process fracture associated with maxillofacial trauma: incidence, distribution, and management. J Oral Maxillofac Surg.

[8] Kermani H, Dehghani N, Aghdashi F, Esmaeelinejad M (2016). Nonsyndromic isolated temporal bone styloid process fracture. Trauma Mon.

[9] Jose A, Nagori SA, Virkhare A, Bhatt K, Bhutia O, Roychoudhury A (2014). Piezoelectric osteoarthrectomy for management of ankylosis of the temporomandibular joint. Br J Oral Maxillofac Surg.

